# Supplementary material for: Molecular maps of diseases from omics data and network embeddings
Source: NPJ Syst Biol Appl. 2026 May 22;12:122. doi: 10.1038/s41540-026-00746-8 (PMC13470450; doi:10.1038/s41540-026-00746-8)
Supplement: Supplementary file 1 [file 41540_2026_746_MOESM1_ESM.pdf]

Supplementary material for: Molecular maps of diseases from omics data and network embeddings

Dataset

Disease	Omics	Positives	Negatives
Atopic dermatitis	GWAS and RNA-seq integration	1000	15622
Ulcerative colitis	GWAS and RNA-seq integration	1300	14422
Focal epilepsy	RNA-seq	1106	18516
Colorectal cancer	Somatic mutation	82	19540
Lymphoma	Somatic mutation	86	19536
Melanoma	Somatic mutation	83	19539
Aortic aneurysm	Proteomics	109	1849

Table S1. Training set size. *Dataset composition showing the number of positive and negative protein examples used to train logistic regression models for each of the seven diseases, along with the corresponding omics data type used.*

Disease	Positives	Negatives
Atopic dermatitis	188	188
Ulcerative colitis	98	98
Focal epilepsy	121	121
Colorectal cancer	49	49
Lymphoma	247	247
Melanoma	404	404
Aortic aneurysm	98	98

Table S2. Test set size. *Composition of balanced literature-based gold standard test sets used for model evaluation, with equal numbers of positive and negative protein examples for each disease.*

		Jaccard Index
Atopic dermatitis	Ulcerative colitis	0.06
Colorectal cancer	Lymphoma	0.07
Colorectal cancer	Melanoma	0.13
Melanoma	Lymphoma	0.09

Table S3. Jaccard index of the training set. *Pairwise similarities between positive training protein sets for different diseases, showing low overlap (0.06-0.13) that validates the diseases have largely distinct associated protein sets despite potential shared mechanisms.*

Cross-validation curves

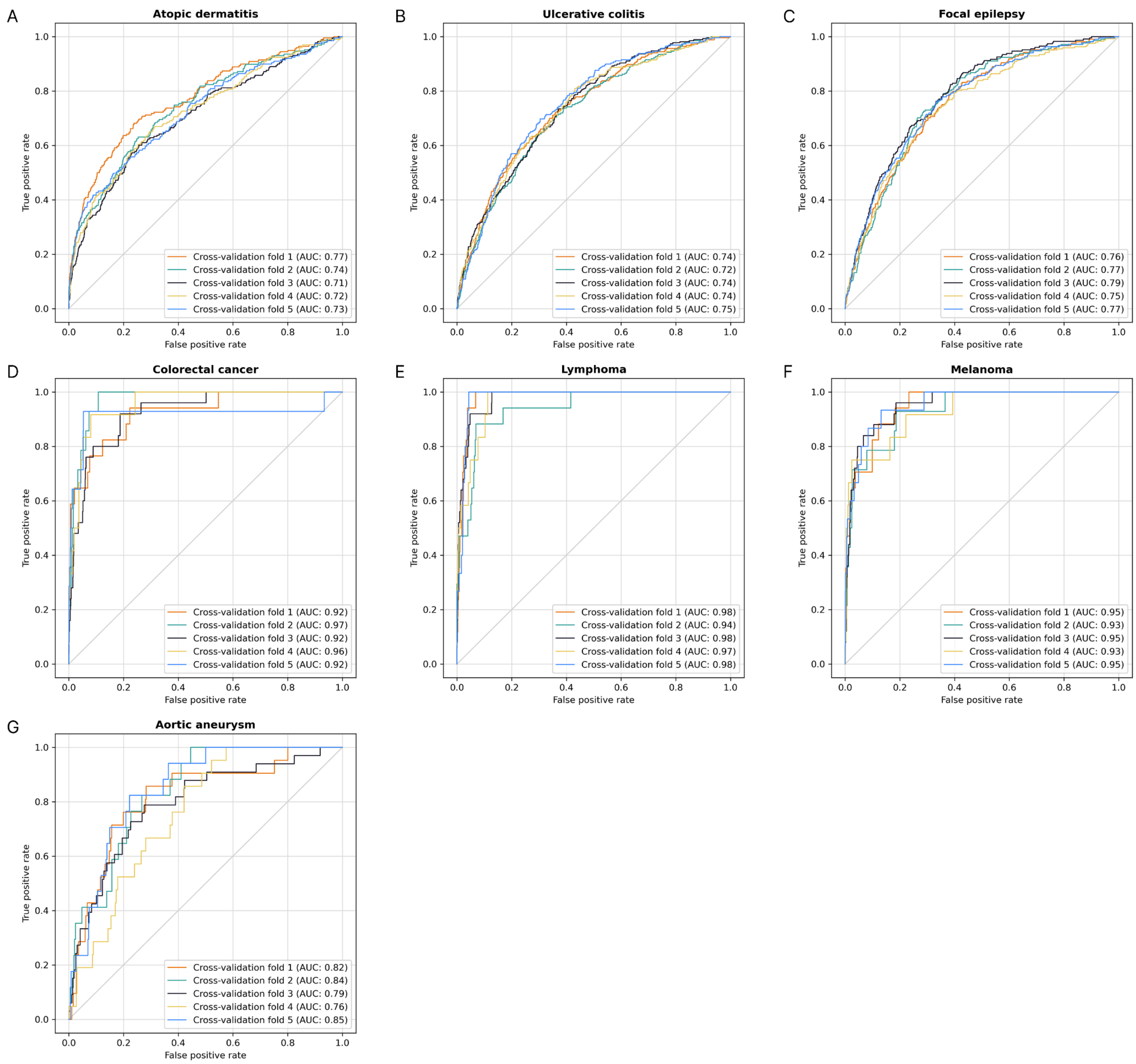

Figure S1. Five-fold cross-validation of the network-based AI method. *(A) Atopic dermatitis. (B) Ulcerative colitis. (C) Focal epilepsy. (D) Colorectal cancer. (E) Lymphoma. (F) Melanoma. (G) Aortic aneurysm. ROC curves for each of the five cross-validation folds across all seven diseases (A-G), with AUC scores labeled for individual folds to demonstrate robustness of the network-based AI method.*

DeLong test of the ROC curves

Method 1	Method 2	P-value
Network-based AI (AUC: 0.95)	GWAS (AUC: 0.74)	4.54×10^{-13}
Network-based AI (AUC: 0.95)	RNA-seq (AUC: 0.64)	7.39×10^{-24}
Network-based AI (AUC: 0.95)	Integrated omics (AUC: 0.80)	3.72×10^{-09}

Table S4. DeLong test results on the atopic dermatitis literature-based gold standard. *Statistical comparison of ROC curve areas showing the network-based AI method significantly outperforms GWAS alone, RNA-seq alone, and integrated omics approaches ($P < 10^{-8}$).*

Method 1	Method 2	P-value
Network-based AI (AUC: 0.95)	GWAS (AUC: 0.65)	$1{,}71\times10^{-25}$
Network-based AI (AUC: 0.95)	RNA-seq (AUC: 0.69)	1.50×10^{-21}
Network-based AI (AUC: 0.95)	Integrated omics (AUC: 0.73)	1.63×10^{-17}

Table S5. DeLong test results on the ulcerative colitis literature-based gold standard. *Statistical comparison of ROC curve areas showing the network-based AI method significantly outperforms GWAS alone, RNA-seq alone, and integrated omics approaches ($P < 10^{-16}$).*

Number of predicted disease-associated proteins

Disease	Total proteins	Positive in omics data	Positive in text mining	Positive in both	Novel predcitions
Atopic dermatitis	1717	370	157	68	1258
Ulcerative colitis	2647	448	160	50	2089
Focal epilepsy	1329	280	67	13	995
Colorectal cancer	2213	76	46	13	2104
Lymphoma	3183	85	266	53	2885
Melanoma	3320	81	236	12	3015
Aortic aneurysm	996	67	45	7	891

Table S6. Number of predicted disease-associated proteins. *Breakdown of total predicted proteins by evidence source, showing proteins identified in omics data, text mining, both omics data and text mining, and novel predictions without prior evidence in each disease.*

Log-weighted degree distribution of positive proteins across diseases

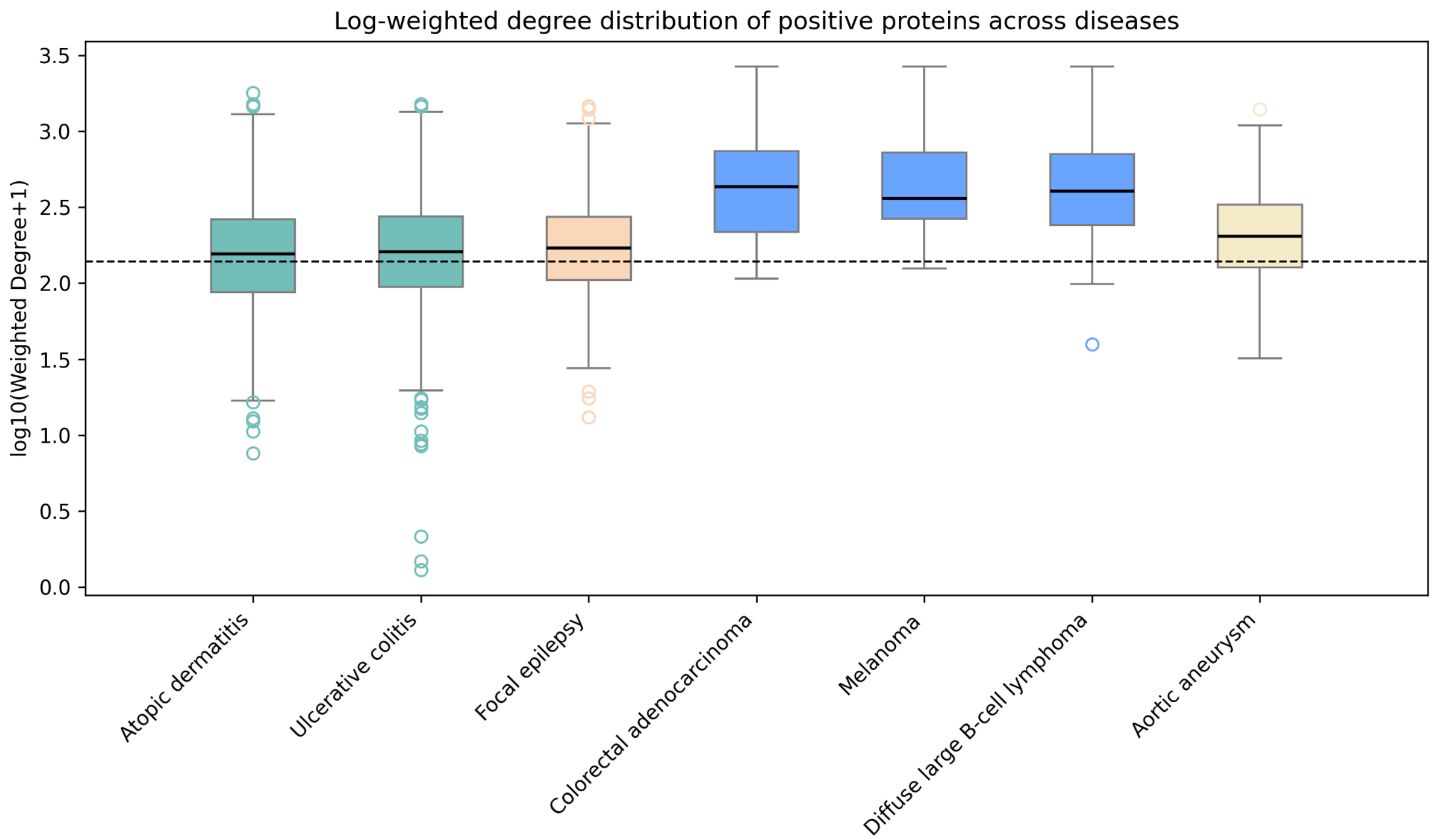

Figure S2. Log-weighted degree distribution of gold-standard positive proteins in the STRING network. *Boxplots show the distribution of log_{10}-transformed weighted degree (log_{10}(weighted degree + 1)) for positive proteins in each disease gold-standard set, where weighted degree is the sum of combined interaction scores across all partners in the STRING human network (see Methods). Cancer-related targets (colorectal adenocarcinoma, melanoma, and diffuse large B-cell lymphoma; blue) exhibit notably higher median degrees than inflammatory (teal), neurological (peach), and vascular (yellow) disease targets. The dashed horizontal line indicates the median log-weighted degree across all human proteins in STRING (2.14).*

Disease mention proportion for gold standard target proteins in the literature

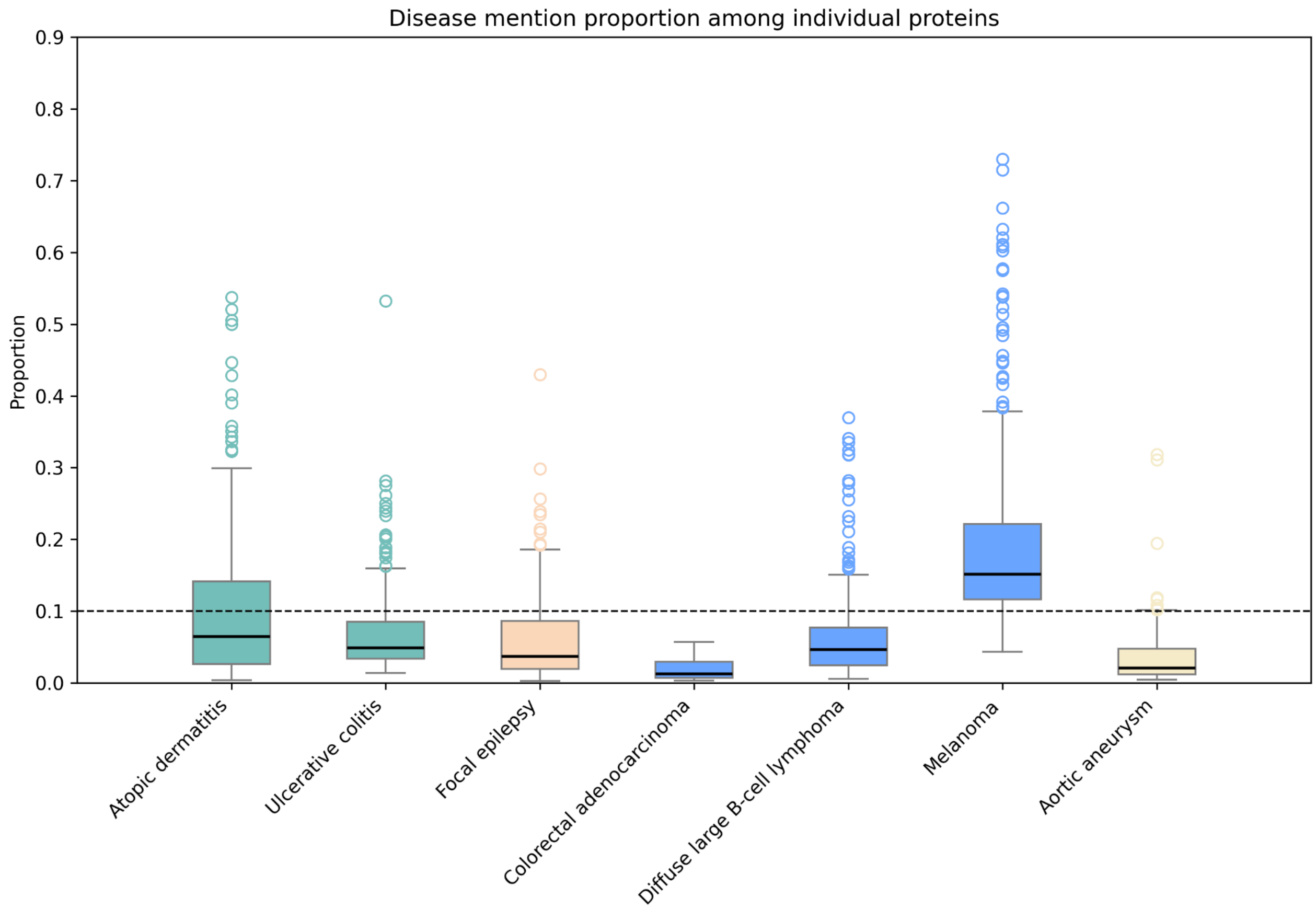

Figure S3. Disease mention proportion for gold standard target proteins in the literature. *Disease mention proportion among individual proteins, defined as the fraction of publications mentioning a given protein that also mention the corresponding disease. The dashed line indicates a proportion of 0.1. Diseases are colored by categories:* inflammatory *(teal), neurological (peach), oncological (blue), and* vascular *(yellow).*

STRING text mining contribution in gold-standard positive proteins

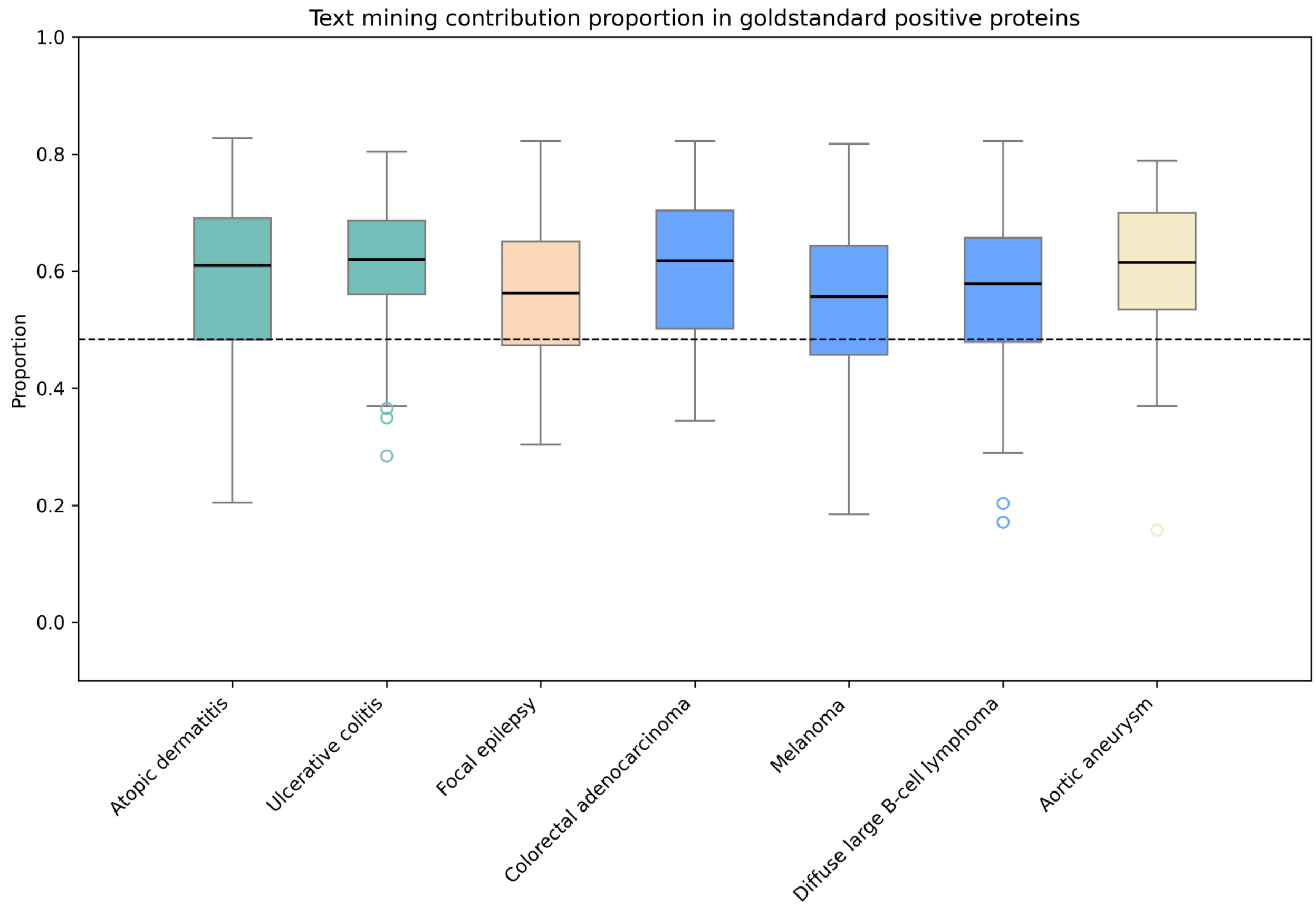

Figure S4. Text mining contribution to STRING combined scores among gold-standard positive proteins. Boxplots show the distribution of the text-mining channel's contribution proportion to the combined interaction score for each positive gold-standard protein, grouped by disease. For each protein, the proportion is defined as (Σcombined scores − Σno-text-mining scores) / Σcombined scores, summed over all interactions involving that protein in the STRING human network. Box colors denote disease categories: inflammatory (teal), neurological (peach), cancer (blue), and vascular (yellow). The dashed horizontal line indicates the median text-mining contribution proportion across all human proteins in STRING (0.483).

Pairwise comparison of log-transformed weighted degrees

Disease1	Disease2	P-value	U-statistic
Atopic dermatitis	Ulcerative colitis	0.2704	630003.0
Atopic dermatitis	Focal epilepsy	0.0023	509442.5
Atopic dermatitis	Colorectal adenocarcinoma	$<10^{-300}$	16349.0
Atopic dermatitis	Melanoma	$<10^{-300}$	14153.0
Atopic dermatitis	Diffuse large B-cell lymphoma	$<10^{-300}$	16638.0
Atopic dermatitis	Aortic aneurysm	0.0002	42750.0
Ulcerative colitis	Focal epilepsy	0.0303	679366.5
Ulcerative colitis	Colorectal adenocarcinoma	$<10^{-300}$	21367.0
Ulcerative colitis	Melanoma	$<10^{-300}$	18453.5
Ulcerative colitis	Diffuse large B-cell lymphoma	$<10^{-300}$	21596.5
Ulcerative colitis	Aortic aneurysm	0.0007	56824.0
Focal epilepsy	Colorectal adenocarcinoma	$<10^{-300}$	19077.5
Focal epilepsy	Melanoma	$<10^{-300}$	16162.5
Focal epilepsy	Diffuse large B-cell lymphoma	$<10^{-300}$	19136.0
Focal epilepsy	Aortic aneurysm	0.0093	51143.5
Colorectal adenocarcinoma	Melanoma	0.6957	3282.5
Colorectal adenocarcinoma	Diffuse large B-cell lymphoma	0.9342	3552.5
Colorectal adenocarcinoma	Aortic aneurysm	$<10^{-300}$	6602.0
Melanoma	Diffuse large B-cell lymphoma	0.8075	3647.0
Melanoma	Aortic aneurysm	$<10^{-300}$	6960.5
Diffuse large B-cell lymphoma	Aortic aneurysm	$<10^{-300}$	7017.0

Table S7. Pairwise Mann-Whitney U test of log-transformed weighted degrees. *The results quantitatively confirm that the higher network connectivity of cancer targets is statistically significant when compared to the targets of inflammatory, neurological, and cardiovascular diseases.*

Functional modules summary

Disease	Total Network Size (Nodes)	Number of Modules (≥3 nodes)	Mean Module Size	Examples
Atopic dermatitis	1717	157	37.89	Keratinization<br>Skin barrier development<br>Inflammatory response
Ulcerative colitis	2647	204	29.05	Cell-cell junction organization<br>Chemokine signaling<br>Immune response
Focal epilepsy	1329	70	10.56	Synaptic signaling<br>Ion transmembrane transport<br>Neurotransmitter regulation
Colorectal adenocarcinoma	2213	158	22.39	Wnt signaling pathway<br>Cell cycle regulation<br>Cellular senescence
Melanoma	3320	33	26.85	Pigmentation<br>Cell population proliferation<br>Kinase signaling
Diffuse large B-cell lymphoma	3183	18	20.44	B cell receptor signaling<br>Apoptotic process<br>Lymphocyte activation
Aortic aneurysm	996	31	11.90	Extracellular matrix organization<br>Collagen fibril organization<br>Smooth muscle contraction

Table S8. Functional modules summary. *The table summarizes the topological and functional metrics of the predicted disease-associated networks. "Total Network Size" indicates the number of top predicted proteins for each condition. "Number of Modules" denotes the total count of significantly enriched Gene Ontology (GO) Biological Process terms (requiring both $P < 0.05$ and $FDR < 0.05$) that contain at least three predicted genes. "Mean Module Size" represents the average number of genes mapped to these significantly enriched GO terms. The "Examples" column highlights representative functional processes identified within each disease network.*

Comparison of functional and physical network embeddings

Disease	Fold	Physical	Functional
Aortic aneurysm	1	0.74	0.82
Aortic aneurysm	2	0.8	0.84
Aortic aneurysm	3	0.72	0.79
Aortic aneurysm	4	0.69	0.76
Aortic aneurysm	5	0.8	0.85
Atopic dermatitis	1	0.65	0.77
Atopic dermatitis	2	0.62	0.74
Atopic dermatitis	3	0.63	0.71
Atopic dermatitis	4	0.68	0.72
Atopic dermatitis	5	0.67	0.73
Colorectal adenocarcinoma	1	0.87	0.92
Colorectal adenocarcinoma	2	0.84	0.97
Colorectal adenocarcinoma	3	0.84	0.93
Colorectal adenocarcinoma	4	0.89	0.96
Colorectal adenocarcinoma	5	0.9	0.91
Diffuse large B-cell lymphoma	1	0.89	0.98
Diffuse large B-cell lymphoma	2	0.9	0.94
Diffuse large B-cell lymphoma	3	0.91	0.97
Diffuse large B-cell lymphoma	4	0.91	0.96
Diffuse large B-cell lymphoma	5	0.93	0.98
Focal epilepsy	1	0.66	0.76
Focal epilepsy	2	0.66	0.77
Focal epilepsy	3	0.66	0.79
Focal epilepsy	4	0.66	0.75
Focal epilepsy	5	0.66	0.76
Melanoma	1	0.75	0.95
Melanoma	2	0.81	0.93
Melanoma	3	0.8	0.95
Melanoma	4	0.86	0.93
Melanoma	5	0.79	0.96
Ulcerative colitis	1	0.68	0.74
Ulcerative colitis	2	0.65	0.72
Ulcerative colitis	3	0.65	0.74
Ulcerative colitis	4	0.63	0.74
Ulcerative colitis	5	0.64	0.75

Table S9. Cross-validation AUC comparison of functional and physical network embeddings. *Functional embeddings consistently outperformed physical network embeddings on all splits and diseases.*

Disease	Physical	Functional
Aortic aneurysm	0.71	0.88
Atopic dermatitis	0.85	0.95
Colorectal adenocarcinoma	0.8	0.99
Diffuse large B-cell lymphoma	0.93	0.99
Focal epilepsy	0.71	0.89
Melanoma	0.84	0.93
Ulcerative colitis	0.85	0.95

Table S10. Test set AUC comparison of functional and physical network embeddings. *Functional embeddings consistently outperformed physical network embeddings on all diseases.*
